# China's groundwater situation in 2011–2020: Dynamic evolution, Influencing Factors, and sustainable development

**DOI:** 10.1016/j.heliyon.2024.e34097

**Published:** 2024-07-04

**Authors:** Huishi Du

**Affiliations:** College of Geographic Science and Tourism, Jilin Normal University, Siping 136000, China

**Keywords:** Groundwater depth, Groundwater development volume, Space-time evolution, Influencing factors, Sustainable development, China

## Abstract

Groundwater plays important roles in resource security and ecological maintenance and is sensitive to changes in the natural environment and human activities. It is of paramount importance to investigate the dynamic evolution trend of groundwater and its affecting factors and sustainable development. This paper takes mainland China as the study area, using data sourced from the *Groundwater Dynamic Monthly Report*, the *China Water Resources Bulletin*, and the *Provincial Water Resources Bulletin*. The temporal and space-time evolution trend of groundwater depth in 2011–2020 is determined, along with the correlation between variations in groundwater resources and precipitation, the factors affecting these changes, and the sustainability of groundwater use. The results are as follows: (1) The northern and western regions of mainland China had a greater depth of groundwater compared to the southern and eastern regions. The largest groundwater depth is in the Northwest Rivers Basin (Nw RB), which can reach 17.61–21.10 m, and the shallowest groundwater depth is in the Southeast Rivers Basin (Se RB), only 1.61–5.19 m. (2) Regarding the factors affecting the changes in groundwater resources, precipitation, land use pattern, human activities, and industrial and agricultural water use are highlighted. (3) Overall, the percentage of groundwater in the total water supply has declined. The optimization of groundwater resource allocation and the adjustment of industrial structure have resulted in the coordinated utilization of groundwater resources. The research establishes a scientific foundation for ensuring national water security and promoting sustainable economic and social development.

## 1 Introduction

As an important water resource, groundwater is an important support for human life and production development, and is greatly affected by human activities and environmental changes [1]. Groundwater refers to water that is stored in the pores of rocks below the ground surface, narrowly defined as water in the saturated aquifer below the water table. The exponential growth of the population and the expansion of metropolitan areas have led to a significant surge in the need for water resources [2,3]. This has resulted in issues such as the overexploitation of groundwater and groundwater pollution. According to statistics, 1 % of the world's groundwater is extracted every year, which has led to an increasing scarcity of many groundwater resources, and more regions and countries have the problem of poor or even depletion of groundwater [4,5]. Recently, the dynamic changes of groundwater have become a hot topic of global research [6,7]. Groundwater is an important ecological factor, and its changes will affect the natural balance of the ecosystem [8]. Excessive groundwater and elevated groundwater level cause secondary salinization of soil and affect plant growth. Excessive exploitation of groundwater has reduced the groundwater level, degraded wetlands, disappeared aquatic plants, damaged the landscape, and even turned the oasis into a desert. China's groundwater resources constitute approximately 33 % of the country's total water resources, making groundwater an important water supply source in regions with a lack of surface water resources [9,10]. Groundwater also plays an irreplaceable role in ensuring economic construction and supporting sustainable economic and social development [11,12].

In January 2012, the State Council put forward the goal of implementing the most rigorous management system, namely the “Opinions on the Implementation of the Strictest Water Resources Management System”, with the aim to clarify the goals and tasks of groundwater treatment in the near future, establish a red line of water resources development and use control, enhance the regulation and administration of groundwater exploitation and groundwater level, and control the amount of sewage discharged into rivers and lakes. In this context, research on the characteristics of space-time changes in groundwater depth is not only practically significant for the development and use of national water resources but also crucial to China's economic development and ecological civilization construction.

Most recent studies focused on the evolution law of surface water resources, the amount of groundwater resources, and their influencing factors. For instance, Yi et al. [13] monitored the reserves of groundwater resources based on GRACE satellites data and comprehensively estimated the changes in groundwater reserves in various river basins in China from 2003 to 2014. Chen et al. [14] systematically analyzed the influencing factors and evolution of China's groundwater resources since 1956. However, studies on the dynamic evolution of the groundwater level at the national level are still scarce as most studies focused on a certain basin or region. An et al. [15] studied the dynamic features of the groundwater level in the Yellow River Delta from 1995 to 2009, whereas Yao et al. [16] investigated the space-time change law of groundwater depth based on monthly groundwater depth data from 1980 to 2007 from the Huaibei Plain. Ding et al. [17] explored the groundwater level change law and its driving factors in the Shihezi-Changji area from 2016 to 2020. Although these studies have contributed to a better understanding of the dynamic changes of the groundwater depth in some parts of China, more research is necessary at the national level. Scholars have confirmed that groundwater resources changes are related to natural and human factors [2,4]. Natural factors are mainly atmospheric precipitation, surface water seeping into the ground, lateral infiltration of the upstream of groundwater, downstream runoff, evaporation of groundwater diving layer, spring water gushing out and groundwater flow to the canal [6]. Human factors include artificial recharge of groundwater, population for industrial water, domestic water and agricultural water. Among the natural factors, atmospheric precipitation is the main influencing factor affecting the change of groundwater reserves [18].

Despite of its vast territory and abundant water resources, China has only a quarter of the amount of water resources per person compared to the world average [4]. With water resources becoming a constraint of social and economic sustainable development, and China has been recognized by the United Nations as a "water resources shortage" country [19,20], requiring improved groundwater management and protection strategies. Based on the *Groundwater Dynamic Monthly Report*, published by the Ministry of Water Resources, the China and provincial *Water Resources Bulletin*, and other relevant reports [4,7]. This study examines the dynamic trends in groundwater depth evolution across China and various water resources subdistricts from 2011 to 2020, determines the characteristics of groundwater resources development, and explores the main factors influencing groundwater changes and sustainable groundwater management. This study not only provides a reference basis for groundwater development, utilization and governance protection, but also provides data support for national water safety and healthy economic and social development.

## 2 Study area

**Fig. 1:**
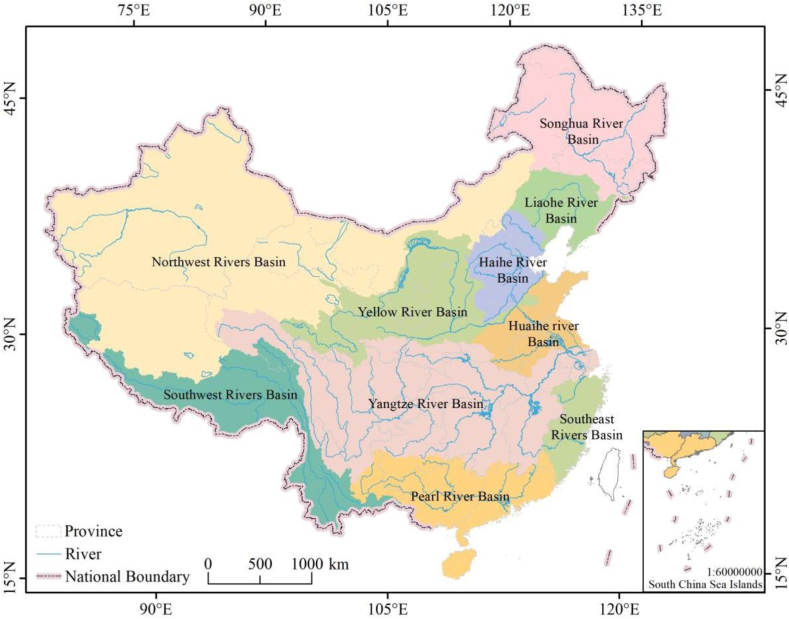
Water resources regionalization of China.

**Fig. 2:**
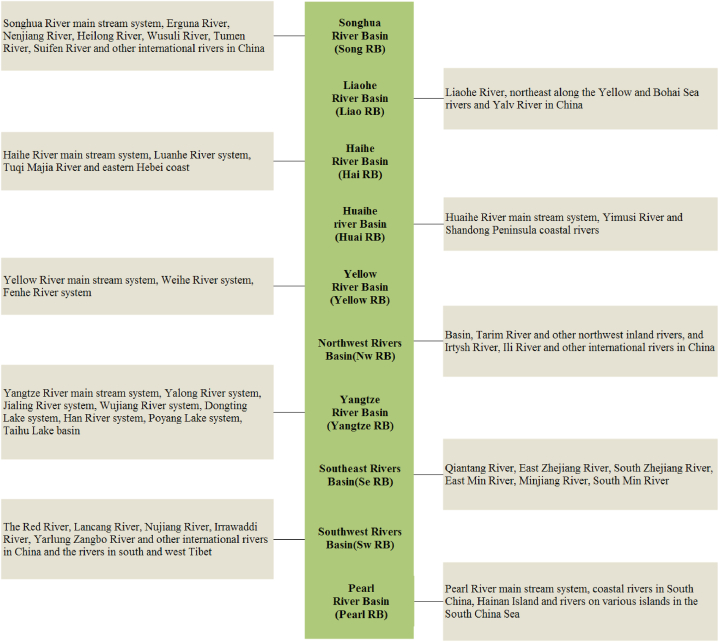
Rivers included in different China's water resources regionalization [21].

Located in the eastern Eurasian continent and the western coast of the Pacific Ocean, China is the largest country in Asia. It has a wide span from north to south and a long distance from east to west, which explains the country's water resources exhibit difference spatial distribution patterns. Based on the differences in watershed integrity and geographical characteristics, the whole study area was divided into ten representative regions [21] (Fig. 1, Fig. 2). Precipitation decreases from southeast to northwest. The average annual precipitation in the southeast coastal region is 1300–2500 mm, whereas the northwestern region, which located in the depths of the mainland, receives an average annual precipitation of 30–300 mm [12].

China shares of water resources is only a quarter of the global average, as well as more than two-thirds of the country's cities are subjected to water shortages [13]. By the end of 2011, the total groundwater development reached 1109.12 billion m^3^/year, accounting for approximately 18.16 % of the total water resources development [15]. In the north and northwest of China, water shortages are more prominent, and these areas heavily rely on groundwater, which accounts for more than 50 % of the used water. In this context, by combining water resource regionalization with key economic zones and understanding the socio-economic development characteristics of each region, we can adjust the use and management of groundwater resources across distinct zones, according to their development status [17].

## 3 Materials and methods

### 3.1 Data and processing

The amount of groundwater resources referred to in this article pertain to the dynamic water that participates in the water cycle, has a direct hydraulic connection with surface water bodies and local precipitation, and can be updated year by year, that is, the amount of shallow groundwater resources [22]. Data on groundwater resources, groundwater depth, groundwater reserves and groundwater extraction were obtained from the *Groundwater Dynamic Monthly Report* and the *China Water Resources Bulletin*, published by the Ministry of Water Resources of the People's Republic of China from 2011 to 2020, as well as the *Provincial Water Resources Bulletin*, issued by the provincial water conservancy departments (http://www.mwr.gov.cn/). The data uses direct measurement to quantify the amount of groundwater resources. Groundwater mining mainly refers to the total amount of groundwater used by human beings, including water used in industry, agriculture, life, etc. The original data of groundwater comes from the automatic monitoring data of 20,469 groundwater monitoring stations in the national groundwater monitoring project of the Ministry of Water Resources and the Ministry of Natural Resources. This paper selects the data of 10,171 monitoring points completed by the Ministry of Natural Resources. The *China Water Resources Bulletin* does not contain relevant data from Taiwan, Macao, and Hong Kong, these regions were not included in the analysis. The location of the monitoring wells are mainly aquifers with development and utilization value. The diving monitoring wells do not penetrate the submersible bottom plate. There is a good relationship between the target layer and the aquifer in the pressurized water monitoring wells. The depth to groundwater table in this paper refers to the groundwater level depth measured by the monitoring well. The data used in this article comes from the China Water Resources Bulletin, which is the data collected, processed and disclosed by the Ministry of Water Resources. Surface runoff data were obtained from the Catchment Land Surface Model of Global Land Data Assimilation System (GLDAS) with a temporal resolution of 1 d and a spatial resolution of 0.25° × 0.25° for the period 2011–2020. Data on groundwater extraction, such as industrial, agricultural, living and other water consumption, comes from the China Statistical Yearbook (2011–2020) (http://www.stats.gov.cn/sj/ndsj/) issued by the China Bureau of Statistics. China's administrative division vector data were acquired from the standard map service website of the National Bureau of Surveying, Mapping, and Geographic Information (http://bzdt.ch.mnr.gov.cn/).

The software packages Python and Excel were used to carry out the Mann-Kendall trend test on groundwater depth data and to determine the correlation between precipitation and groundwater resources. The graphs were generated using the Origin 8.5 software. Applying the ArcGIS10.8 software, the spatial interpolation of China's groundwater depth from 2011 to 2020 was interpolated, and the zoning of groundwater resources was performed to obtain the spatial change trend of groundwater depth.

### 3.2 Research methods

#### 3.2.1 Mann-Kendall test method

Mann-Kendall (M − K) trend test is a ranking-based, non-parametric method for trend change analysis. It arranges data chronologically and assesses each consecutive datum. This method is used to examine the trend characteristics of climate change and groundwater resource quantity change [[23], [24], [25]]. The trend of the time series is determined by the *U* value, using the following equation:

(1) $$U=\left\{ \begin{array}{c} \frac{\left(S-1\right)}{\sqrt{n\left(n-1\right)\left(2n+5\right)/18}}\;\left(S>0\right)\\ \;0\;\left(S=0\right)\\ \frac{\left(S+1\right)}{\sqrt{n\left(n-1\right)\left(2n+5\right)/18}}\;\left(S<0\right) \end{array}\right.$$

where *U* is the value to determine the trend of the data, *S* represents the statistics of the two sizes of all data, *n* is the number of samples. When n≥10, *S* approximates the normal distribution. For a specified level of confidence *α*, in the context of bilateral trend analysis, if $U>U_{\frac{\alpha }{2}}$, there is a significant upward trend in the sequence; $U<U_{\frac{\alpha }{2}}$ indicates that there is a significant downward trend in the sequence.

The magnitude of the trend change is given by Sen's slope estimate (β):

(2) $$\beta =\text{Median}\left(\frac{x_{i}-x_{j}}{i-j}\right)\left(\forall j<i,1\leq j\leq i\leq n\right)$$

where *i* and *j* represent a specific time on the sequence, $x_{i}$ and $x_{j}$ are the time series data of *i* and *j* time points. If β is positive, the sequence shows an upward trend; if β is negative, it shows a downward trend.

#### 3.2.2 Correlation analysis

Correlation analysis is commonly employed to ascertain the correlation between variables by means of the correlation coefficient, a statistical measure of the degree of association between variables. This paper mainly uses this method to analyze the correlation between underground water storage and precipitation. The correlation coefficient is calculated as follows:

(3) $$r_{xy}=\frac{S_{xy}}{S_{x}S_{y}}$$

where $r_{xy}$ represents the sample correlation coefficient, $S_{xy}$ is the sample covariance, $S_{x}$ represents the sample standard deviation of *x*, and $S_{y}$ represents the sample standard deviation of *y*. The following are the calculation formulas of $S_{xy}$ covariance and $S_{x}$ and $S_{y}$ standard deviation; the denominator uses *n-1*.

$S_{xy}$ sample covariance calculation:

(4) $$S_{xy}=\frac{\sum_{i=1}^{n}\left(X_{i}-\bar{X}\right)\left(Y_{i}-\bar{Y}\right)}{n-1}$$

$S_{x}$ sample standard deviation calculation:

(5) $$S_{x}=\sqrt{\frac{\sum {\left(x_{i}-\bar{x}\right)}^{2}}{n-1}}$$

$S_{y}$ sample standard deviation calculation:

(6) $$S_{y}=\sqrt{\frac{\sum {\left(y_{i}-\bar{y}\right)}^{2}}{n-1}}$$

#### 3.2.3 Kriging interpolation

The groundwater level Kriging interpolation method can be employed to performed to estimate the regional changes of unknown sampling points. It is the most commonly used interpolation method for groundwater level analysis in geological statistics [22]. This paper uses the interpolation method to calculate the groundwater level of the unknown sampling area. The equation is as follows:

(7) $$Z\left(x_{0}\right)=\sum_{i=1}^{n}\lambda_{i}Z\left(x_{i}\right),\sum_{i=1}^{n}\lambda_{i}=1$$

where *Z* is the estimated value of the groundwater level, $x_{i}$ represents the position of the monitoring point, while *n* represents the total number of interpolation points, and $\lambda_{i}$ r is the weight coefficient of each monitoring point in the interpolation process.

## 4 Results

### 4.1 Dynamic evolution of groundwater depth in China

#### 4.1.1 Temporal variation characteristics of groundwater depth

**Fig. 3:**
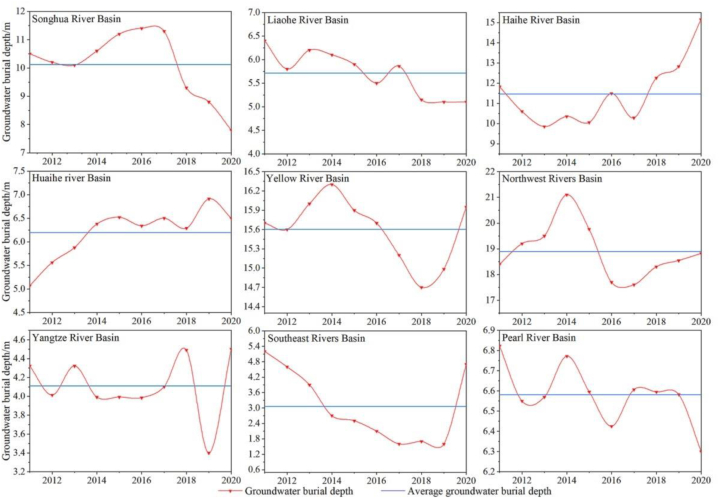
Inter-annual changes in groundwater depth in China's water resources regionalization.

According to the M − K test, the change trend of groundwater depth in different water resources zones in mainland China is analyzed (Table 1). The groundwater depth in most water resources areas in China shows different degrees of change. The groundwater depth in Hai RB, Huai RB and Yangtze RB shows an upward trend. The groundwater depth in Song RB, Liao RB, Yellow RB, Se RB, Pearl RB and Nw RB shows a downward trend. In the water resources zoning where groundwater depth is declining, the downward trend of Liao RB is very significant, and the absolute value of trend U reaches 3.46, which has passed the 99 % reliability significance test, followed by the Pearl RB, and finally Song RB, Yellow RB and Se RB. In the water resources zoning where groundwater depth is on the rise, the upward trend of Hai RB is relatively significant, with the absolute value of trend U reaching 2.11, followed by Huai RB, where the groundwater depth is gradually increasing. The Yangtze RB and the Nw RB did not pass the significant test, indicating that groundwater depth has not changed much. There is a significant difference in the depth of groundwater in different water resources (Fig. 3), Song RB and Huai RB show a trend of first rise and then fall, with a change of 25.71 % and 28.20 % respectively. The Hai RB shows a trend of first decline and then rise, with a change of 28.15 %. Liao RB and Zhu RB are declining almost continuously, with a change of 20.31 % and 7.67 % respectively. The changes in Yellow RB, Yangtze RB, Se RB, Zhu RB and Nw RB are relatively small.

**Table 1:** Characteristics of changes in groundwater depth within different water resource regionalization.

Water resources regionalization	Trend of groundwater depth	Trending U absolute value
Song RB	-*	1.48
Liao RB	-***	3.46
Hai RB	+**	2.11
Yellow RB	-*	1.40
Huai RB	+*	1.32
Yangtze RB	+	0.70
Se RB	-*	1.56
Pearl RB	-**	1.95
Nw RB	–	0.47

Note: *, ** and *** indicate that they have passed the 90 %, 95 % and 99 % of the confidence significance test respectively.

#### 4.1.2 Spatial characteristics of groundwater depth

In mainland China, the average groundwater depth in 2011–2020 was 8.75–9.47 m (Fig. 3 and Table 2). This level was significantly higher in the northern region compared with the southern one, whereas the western region showed higher levels than the eastern one. Generally, the groundwater depth gradually increased from southeast to northwest. The maximum groundwater depth was found for the Nw RB, reaching 17.61–21.10 m, followed by those for the Yellow RB and the Hai RB with 14.72–16.34 m and 9.85–15.23 m, respectively. The lowest levels were observed for the Se RB with 1.61–5.19 m, followed by the Yellow RB with 3.43–4.50 m. The levels for the Song RB, Liao RB, Huai RB, and Pearl RB ranged from 5.00 to 11.00 m.

**Table 2:** Changes in the average depth of groundwater in China's water resources regionalization from 2011 to 2020 (Unit: m).

Water resources regionalization	2011	2012	2013	2014	2015	2016	2017	2018	2019	2020
Song RB	10.5	10.2	10.1	10.6	11.2	11.4	11.3	9.35	8.76	7.78
Liao RB	6.42	5.85	6.21	6.13	5.88	5.53	5.86	5.15	5.09	5.10
Hai RB	11.82	10.6	9.85	10.35	10.05	11.49	10.28	12.16	12.83	15.15
Yellow RB	15.71	15.62	16.00	16.29	15.91	15.72	15.19	14.73	14.98	15.95
Huai RB	5.07	5.56	5.88	6.38	6.52	6.34	6.50	6.29	6.91	6.50
Yangtze RB	4.32	4.01	4.32	3.99	3.99	3.99	4.10	4.49	3.41	4.48
Se RB	5.09	4.57	3.88	2.72	2.49	2.03	1.62	1.71	1.63	5.08
Pearl RB	6.82	6.55	6.57	6.77	6.60	6.43	6.61	6.60	6.58	6.32
Nw RB	18.40	19.19	19.51	21.10	19.76	17.69	17.60	18.31	18.54	18.82

**Fig. 4:**
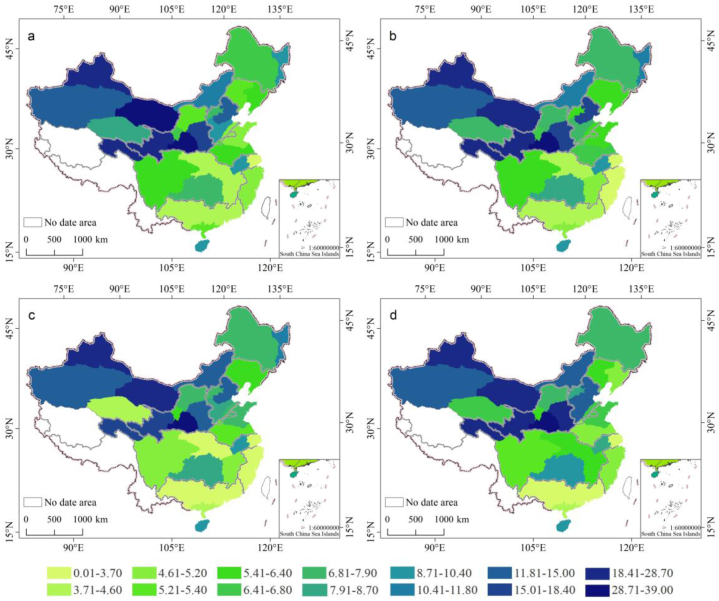
Spatial changes in groundwater depth in China's water resource regionalization.((a) 2011; (b) 2014; (c)2017; (d) 2020).

According to the data of the groundwater level monitoring station released by the Ministry of Water Resources, Kriging interpolation is used to make a difference in the groundwater depth data for 2011, 2014, 2017 and 2020. From 2011 to 2020, the overall groundwater depth in mainland China did not have a significant change, although the spatial changes were considerable (Fig. 4). We observed a significant decrease for the Song RB (2.71 m), the Liao RB (1.32 m), and the Pearl RB (0.52 m). In the Huai RB and the Hai RB, the groundwater level increased considerably, with the largest value for the Hai RB (3.32 m). The Yellow RB and the Nw RB showed an initial increase, followed by a decrease, whereas the Yangtze RB showed the opposite pattern.

### 4.2 Factors influencing the changes in groundwater resources in China

**Fig. 5:**
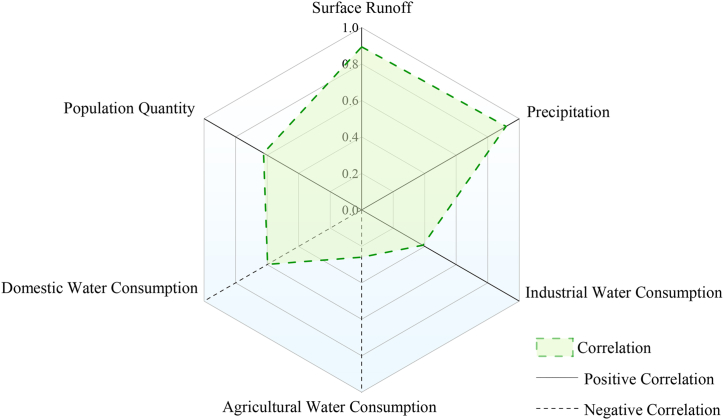
Correlation coefficient between natural and human factors and groundwater resources.

The infiltration of surface water into the ground also has a certain impact on the change of groundwater reserves. Part of the atmospheric precipitation seeps into the underground recharge groundwater reserves, and the other part forms runoff on the surface. Among the human factors, population and domestic water consumption have the most obvious impact on groundwater reserves. The increase in population means an increase in domestic water consumption, especially in northern China, where water resource is relatively scarce and groundwater exploitation is large [4]. Natural factors correlate very significantly with groundwater reserves, with precipitation being the most correlated with a correlation coefficient of 0.92, followed by surface runoff with a correlation coefficient of 0.89. Among the human factors, agricultural water and domestic water are negatively correlated with groundwater reserves, and the number of populations, industrial water, agricultural water, domestic water and groundwater reserves are significantly correlated, which are 0.62, 0.39, −0.60 and −0.26 respectively (Fig. 5).

#### 4.2.1 Natural factors

**Fig. 6:**
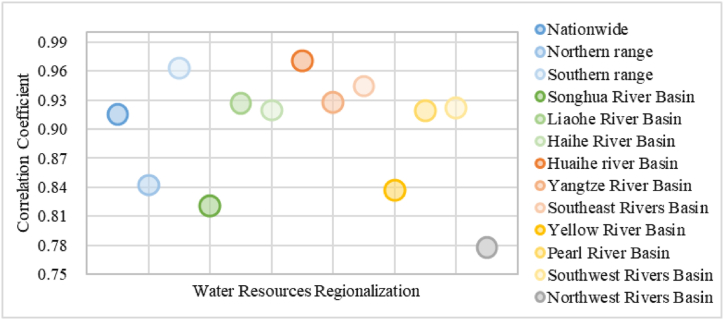
Coefficient diagram of correlation between precipitation and groundwater resources. Note: Northern range refers to the six areas of Yellow RB, Song RB, Hai RB, Liao RB, Huai RB and Nw RB; southern range refers to the four areas of Pearl RB, Yangtze RB, Se RB and Sw RB (For interpretation of the references to colour in this figure legend, the reader is referred to the Web version of this article).

The natural factors affecting the reserves of groundwater resources are mainly due to the direct infiltration of atmospheric precipitation and the infiltration of surface water into the ground. Among them, the supply of atmospheric precipitation directly penetrates into groundwater accounts for about 13.5 %, and the recharge of surface water penetrating into groundwater accounts for about 82.5 % [26]. Regional climate differences and changes lead to changes in precipitation between regions, which will affect the infiltration of atmospheric precipitation and ultimately the amount of groundwater resources. Under suitable geological conditions, in areas with abundant rainfall, groundwater can obtain a large amount of recharge, and groundwater reserves are relatively abundant, while in arid areas, the evaporation of groundwater diving layer is large, and groundwater reserves are scarce [27]. According to the correlation analysis between precipitation and groundwater resources (Fig. 6), the correlation between the inland and the northeast region was lower than that between the coastal and the southern region. The northeast experiences a long and cold winter, and the permafrost layer in the soil impedes water infiltration. In spring, when the temperature rises, the snow melts, and the melting water can infiltrate the soil. The proportion of meltwater from ice and snow in high-altitude mountains was higher than that in the other regions. The inland areas are far away from the sea are not affected by the monsoon and therefore receive less precipitation.

**Fig. 7:**
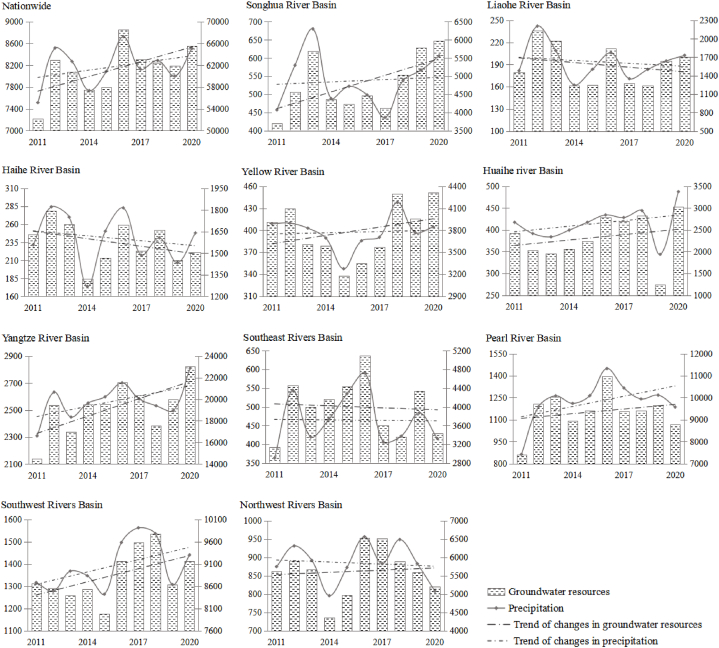
Variation trends of groundwater resources and precipitation in different water resource regionalization.

**Fig. 8:**
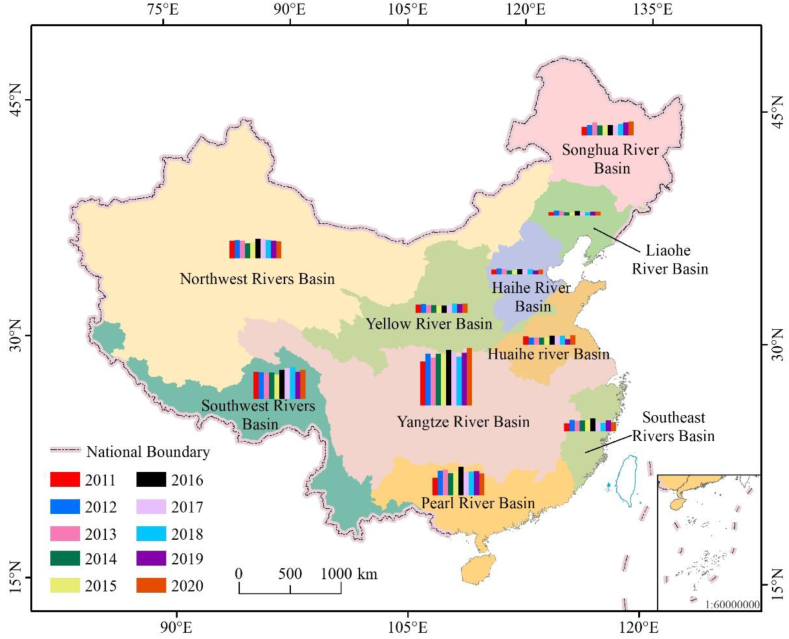
Regional characteristics of groundwater resources in China.

From a national perspective, precipitation fluctuated and increased from 2011 to 2020, which is consistent with the changes of groundwater resources (Fig. 7 and Fig. 8). However, from the perspective of the trend line, both the national precipitation trend line and the groundwater resource trend line show an upward trend. The precipitation trend line from 2011 to 2015 is significantly higher than the groundwater resource trend line, and the precipitation trend line from 2017 to 2020 is significantly lower than the groundwater resource trend line, which shows that China's groundwater resources are gradually increasing and the environment tends to improve. Based on the geographical differences between the northern and southern regions, the difference in precipitation can be up to 10 times. The precipitation and groundwater resources in the Yangtze RB, the Song RB, the Yellow RB the Hai RB, the Pearl RB, and the Sw RB show an upward trend, which is consistent with the change trend of national precipitation and groundwater resources, showing an upward trend. In contrast, the precipitation and groundwater resources in the Se RB, the Hai RB, and the Liao RB showed a downward trend. The precipitation in the Nw RB shows a downward trend, but the amount of groundwater resources is slowly rising. This is because the area is located in Eurasia and has less rainfall. However, in recent years, due to China's great importance on the protection of groundwater, the amount of groundwater extraction has decreased, and the amount of groundwater resources has shown an increasing trend [21].

With the progress of social and economic development, accelerated urbanization, and water and soil conservation programs, China's land use pattern has undergone significant changes. In addition, the national and regional water supply pattern has been gradually improved through cross-basin water diversion projects such as the South-to-North Water Diversion and large-scale irrigation [28,29]. The changes in the land surface resulted in drastic changes, groundwater replenishment and drainage mechanisms have undergone great changes in many parts of the north [30,31]. According to the relationship between precipitation and groundwater resources and the changing trend, the production capacity of the Yellow RB increased significantly during 2011–2020. Although the average annual precipitation between 2011 and 2020 was stable, the amount of groundwater resources showed an increasing trend. The relationship between groundwater resources and precipitation in many areas, such as the Pearl RB, the Yangtze RB, and the Song RB, showed a increasing trend. The relationship between precipitation and water resources in Liao RB and Hai RB shows a downward trend (Fig. 7). This is due to the temperature rises, the atmospheric precipitation decreases, and the soil water evaporation is large. In addition, in recent years, the population of the area has increased, and the groundwater has been irrationally developed and utilized, resulting in a serious shortage of groundwater, which cannot be effectively restored, and the amount of groundwater resources significantly reduced.

#### 4.2.2 Human factors

In terms of groundwater recharge, due to human activities such as well drilling and drainage, the groundwater level in the northern plains decreased considerably [32]. For example, the average depth of groundwater in the Taihang Mountain Plain increased from 3 to 4 m in the 1980s to 30–40 m in recent years [33]. After the groundwater level decreases, precipitation infiltration is prolonged, the precipitation infiltration recharge coefficient decrease, and the water infiltration recharge is significantly reduced [34]. The huge difference in precipitation in different regions leads to differences in the recharge of groundwater resources in different regions, as well as differences in the total amount of groundwater resources. From the relationship and change trend between precipitation and groundwater resources in 2011–2020, it can be seen that the precipitation in Hai RB decreased in 2011–2020, and the groundwater recharge also decreased significantly (Fig. 7). The relationship between precipitation and groundwater recharge in Liao RB and Se RB showed a downward trend. With the increase of population in recent years, in order to meet the needs of human production and life, the farmland area has increased, the amount of irrigation water consumption has increased significantly, and the use of groundwater resources has also increased, further changing the groundwater recharge structure. Compared with the northern China, there are more rivers in the southern China, and the amount of groundwater extracted is relatively small compared with the northern China. The amount of groundwater resources in the southern China accounts for about 2.3%–3.8 % of the total amount of groundwater resources, while the amount of groundwater resources in the northern China can account for about 30.6%–35.5 % of the total amount of groundwater resources [15,19].

## 5 Discussion

### 5.1 China's groundwater and sustainable socio-economic development

**Fig. 9:**
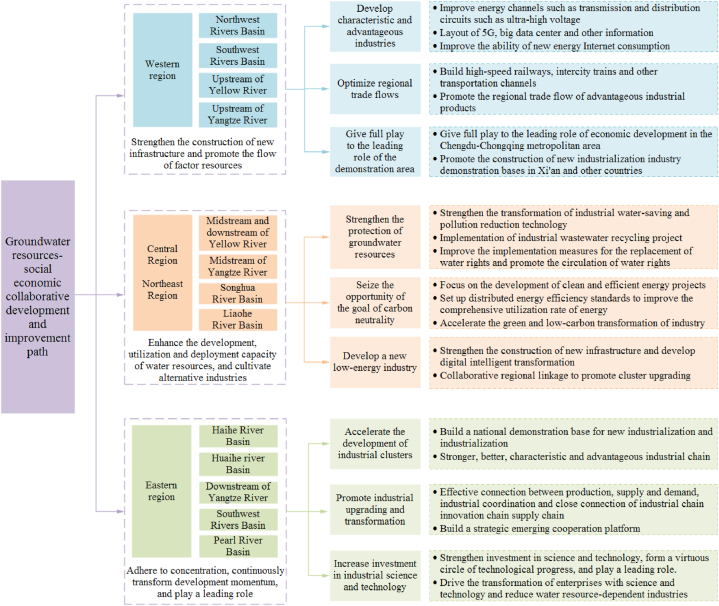
China's regional coordination and high-quality development path.

The groundwater resources-social economic system is a typical open complex giant system. Its virtuous circle is the foundation and prerequisite for social and economic activities and an important guarantee for sustainable social and economic development [[35], [36], [37], [38]]. This study found that between 2011 and 2020, China's groundwater resources fluctuated (Fig. 7). In 2012, 2016 and 2020, China's groundwater resources were relatively large, and 2011–2015 showed a trend of increasing first and then decreasing. In 2016, it reached the maximum value in the past 10 years, which is due to the large precipitation in that year and the better replenishment of groundwater resources. Tu et al. [39] based on the GRACE satellite data, the amount of groundwater resources in China is monitored, and it is found that the amount of groundwater resources in China from 2002 to 2016 is decreasing year by year, and the correlation with the measured data is more than 0.6, and the results are more credible. The results of this study found that although the overall groundwater resources in China have increased, the amount of groundwater resources in Liao RB, Hai RB and Se RB in northern China is still decreasing. In northern China, groundwater exploitation accounts for about 33 % of groundwater resources on average, which is more than 10 times that of southern regions. In northern regions such as Hai RB and Liao RB, groundwater exploitation accounts for more than 50 % of groundwater resources [39]. The research of Mu et al. [40] and Feng et al. [41] also agree with this view. The amount of groundwater exploitation greatly affects the amount of groundwater resources in northern China, and groundwater environmental protection is still facing a severe test. China has a vast territory, so we should optimize the coordinated development of regional industries according to local conditions (Fig. 9).

### 5.2 China's groundwater and the United Nations sustainable development goals

In September 2015, the United Nations Development Summit adopted the " Transforming our World: The 2030 Agenda for Sustainable Development", proposing 17 sustainable development goals (SDGs) and 169 specific indicators [42,43]. China proposed an action to build a "Beautiful China" in response to the SDGs [44]. Among them, water resources-related indicators are a crucial part of evaluating the effectiveness of the "Beautiful China" construction and the realization of SDGs. For water resources, SDG6 proposes to significantly improve water efficiency and reduce water shortages by 2030. The above goals correspond to the economic sustainability of water resource use, ensuring water safety. The above goals correspond to the social sustainability of water resource use. China already made continuous efforts in groundwater protection and governance. For instance, Feng et al. [41] found a reinforced decoupling trend between China's groundwater water supply and economic development. This finding agrees with the outcomes of this research. The proportion of groundwater in the total water supply in the Yellow RB, the Hai RB, the Song RB, the Huai RB and the Liao RB gradually decreased. The largest decrease was observed for the Hai RB, from 63.48 % in 2011 to 39.73 % in 2020. This basin has a developed industry and therefore a large water demand; the decrease in the groundwater proportion reflects the remarkable achievements in groundwater treatment.

Shen et al. [45] and Huang et al. [46] investigated the current status of groundwater resource development and use in t the Pearl RB and the Yangtze RB, respectively, obtained similar values for these two basins. The Se RB, the Pearl RB, the Yangtze RB and the Sw RB are rich in surface water resources, and groundwater exploitation is therefore low. Qin et al. [47], predicted an increase in groundwater utilization based on their analysis of the use and development potential of groundwater resources in arid regions of northwest China. In contrast, Zhou et al. [48] state that the amount of groundwater on the Qinghai-Tibet Plateau decreased significantly during1997–2018. The Nw RB located in the depths of the mainland, with less precipitation and a lack of surface water; consequently, the use of groundwater is increasing (Table 3). Based on the different environmental characteristics and water use structure of each region in China, a more detailed integrated water resources management system should be developed to solve a series of problems arising from water resources development [[49], [50], [51]]. Strengthening the sustainable use and management of groundwater resources in China is an important part of China's actions to achieve SDG6.

**Table 3:** The proportion of groundwater development in China's total water supply.

Water resources regionalization	2011	2012	2013	2014	2015	2016	2017	2018	2019	2020
Song RB	40.57 %	42.42 %	42.91 %	43.04 %	41.26 %	43.32 %	42.68 %	41.40 %	40.18 %	37.43 %
Liao RB	52.56 %	50.87 %	50.37 %	50.63 %	50.44 %	51.60 %	51.58 %	52.66 %	51.78 %	49.84 %
Hai RB	63.48 %	62.32 %	60.56 %	59.31 %	56.47 %	53.70 %	50.00 %	47.21 %	42.25 %	39.73 %
Yellow RB	31.90 %	33.58 %	32.35 %	32.18 %	31.33 %	31.07 %	30.06 %	29.92 %	28.56 %	28.14 %
Huai RB	27.08 %	28.02 %	27.52 %	25.33 %	26.19 %	25.66 %	24.72 %	24.38 %	23.09 %	23.50 %
Yangtze RB	3.97 %	4.03 %	3.82 %	4.04 %	3.48 %	3.37 %	3.27 %	2.99 %	2.90 %	2.06 %
Se RB	2.54 %	2.85 %	2.54 %	2.47 %	2.14 %	2.08 %	1.82 %	1.61 %	1.72 %	1.22 %
Pearl RB	4.11 %	4.01 %	3.91 %	3.84 %	3.81 %	3.75 %	3.58 %	3.40 %	3.28 %	3.09 %
Sw RB	3.34 %	3.89 %	4.35 %	4.82 %	3.61 %	3.13 %	4.00 %	3.85 %	3.99 %	3.96 %
Nw RB	20.47 %	20.32 %	21.43 %	24.02 %	22.40 %	22.61 %	21.70 %	20.51 %	20.09 %	23.35 %

### 5.3 Opportunities and challenges

China is a country facing water scarcity, with its per capita share of water resources only amounting to one quarter of the global average [5]. According to the statistics in 2020 from the Ministry of Water Resources, more than 400 of China's 669 cities are subjected to water shortages, albeit at different degrees, and 110 experience serious water shortages. Especially in northern and northwestern China, water shortages are more prominent. Based on data released by the Ministry of Ecology and Environment, China's wastewater output in 2020 was 571.36 billion m^3^. Most of the domestic sewage is still discharged into rivers or directly used for irrigation without adequate treatment. Currently, in China's social and economic development, groundwater resources are generally overused, and the conflict between supply and demand has intensified, with serious impacts on environmental and human health. In many cities, water pollution has led to water supply issues, posing a risk to human health. Although the ecological degradation of groundwater has, to some extent, been alleviated, the ecological functions in some areas are still impaired. In particular, the large-scale exploitation of mineral resources and engineering activities have brought a series of disastrous groundwater problems. At the same time, China's groundwater situation is also facing many new situations, such as the new economic normal, the pursuit of the new momentum, urbanization, and industrialization, ecological civilization and green development, the response to climate change, and biodiversity protection.

### 5.4 Suggestions for the adequate use of groundwater resources in China

Compared with the United States and other Western countries, groundwater research in China still have significant gaps. However, the theoretical and practical application of China's groundwater science has made great achievements in the past 50 years. To improve the research level of China's groundwater science, it is still necessary to further improve the problems in the development process. Firstly, we should strengthen support for scientific research projects in the field of groundwater science and encourage scholars to conduct long-term and in-depth research on groundwater science issues. Secondly, it is urgent to strengthen the basic research of groundwater science in China and combine field experiments with indoor experiments to provide key raw data for groundwater research. Thirdly, strengthen substantive cooperation among scientists in different contexts and conduct comprehensive research on groundwater and surface water. Fourthly, strengthening groundwater monitoring and data sharing, and providing high-quality and standardized groundwater simulation software is of great significance to groundwater research. Finally, it is necessary to conduct a careful analysis to clarify the gap between hydrogeological education in China and foreign developed countries, and cultivate a group of first-class scholars with a global vision in this field.

## 6 Conclusions

Groundwater depth in mainland China is high in the north and west and low in the south and east. The largest groundwater depth was observed for the Nw RB, reaching 17.61–21.10 m, and the lowest level was found for the Se RB with 1.61–5.19 m. In the Pearl RB, the Liao RB, the Song RB and the Se RB, the groundwater situation improved, whereas in the Huai RB and the Hai RB, the situation worsened. The groundwater resources were distributed unevenly, and the multi-year averages of the Yangtze RB, the Sw RB, and the Pearl RB were 2519.85, 1349.41, and 1156.60 billion m^3^, respectively.

The change of groundwater resources is closely related to natural factors such as the increase or decrease of precipitation and surface water seepage. The change of land use pattern, the increase of industrial and agricultural water use, well drilling, dredging and drainage and other human activities are important factors leading to the change of groundwater reserves. The exploitation of groundwater resources by human activities has gradually increased, but atmospheric precipitation and natural factors of surface water seepage are still the main recharge of groundwater resources, and the impact of atmospheric precipitation on groundwater resources is more significant.

From 2011 to 2020, the amount of groundwater resources in China has shown an upward trend. The amount of groundwater exploitation in Song RB, Liao RB, Hai RB, Yellow RB and Huai RB has gradually decreased. Among them, the decline in Hai RB is the largest, with a decrease of 23.75 %. Yangtze RB, Pearl RB, Se RB and Sw RB are rich in surface water resources, and the amount of groundwater resources is not large. Generally, the groundwater situation has improved, and groundwater use has become more sustainable.

Groundwater dynamic changes are continuous and complex. Since the article is based on bulletin data, it is difficult to obtain smaller-scale data due to factors such as traffic accessibility, coupled with the lack of groundwater depth data in the Sw RB, the results may be unreliable. Further studies will have to take these limitations into account and obtain more complete, detailed, and objective datasets, and integrate GRACE data to improve the quality of spatial interpolation, which can more accurately assess the sustainability of groundwater at the national scale.

## Availability of data and materials

The data used and/or analyzed during this study are available from the corresponding author on reasonable request.

## CRediT authorship contribution statement

**Huishi Du:** Writing – original draft.

## Declaration of competing interest

The authors declare that they have no known competing financial interests or personal relationships that could have appeared to influence the work reported in this paper.
